# Tocilizumab‐induced cytomegalovirus colitis in a patient with COVID‐19

**DOI:** 10.1002/ccr3.3487

**Published:** 2020-11-12

**Authors:** Mohamad Y. Khatib, Karimulla S. Shaik, Amna A. Ahmed, Mohammad A. Alwraidat, Ahmed S. Mohamed, Mohamad R. Abou Kamar, Mouhammad Z. Sharaf Eldean, Bashar K. Aldaraiseh, Abdulqadir J. Nashwan

**Affiliations:** ^1^ Critical Care Unit Hazm Mebaireek General Hospital (HMGH) Hamad Medical Corporation (HMC) Doha Qatar; ^2^ Department of Infectious Diseases Communicable Disease Center (CDC) Hamad Medical Corporation (HMC) Doha Qatar; ^3^ Department of Laboratory Medicine and Pathology (DLMP) Hamad Medical Corporation (HMC) Doha Qatar

**Keywords:** COVID‐19, cytomegalovirus colitis, double‐barrel colostomy, lower gastrointestinal bleeding, tocilizumab

## Abstract

The authors urge clinicians to observe for early signs of CMV reactivation in patients presenting with gastrointestinal bleeding and intestinal perforation after receiving tocilizumab or other immunosuppressive therapy as a treatment for COVID 19. Early recognition of CMV infection and treatment will prevent life‐threatening bleeding and mortality.

## Graphical abstract

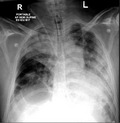

## 1 BACKGROUND

Cytomegalovirus exists in 50%‐80% of the world population in clinically undetected form due to their immunocompetent status. We describe a case of COVID‐19 pneumonia on tocilizumab presenting with massive lower gastrointestinal bleeding due to Cytomegalovirus colitis confirmed by histopathology and serology. The performance of life‐saving hemicolectomy saved our patient.

**Figure 1:**
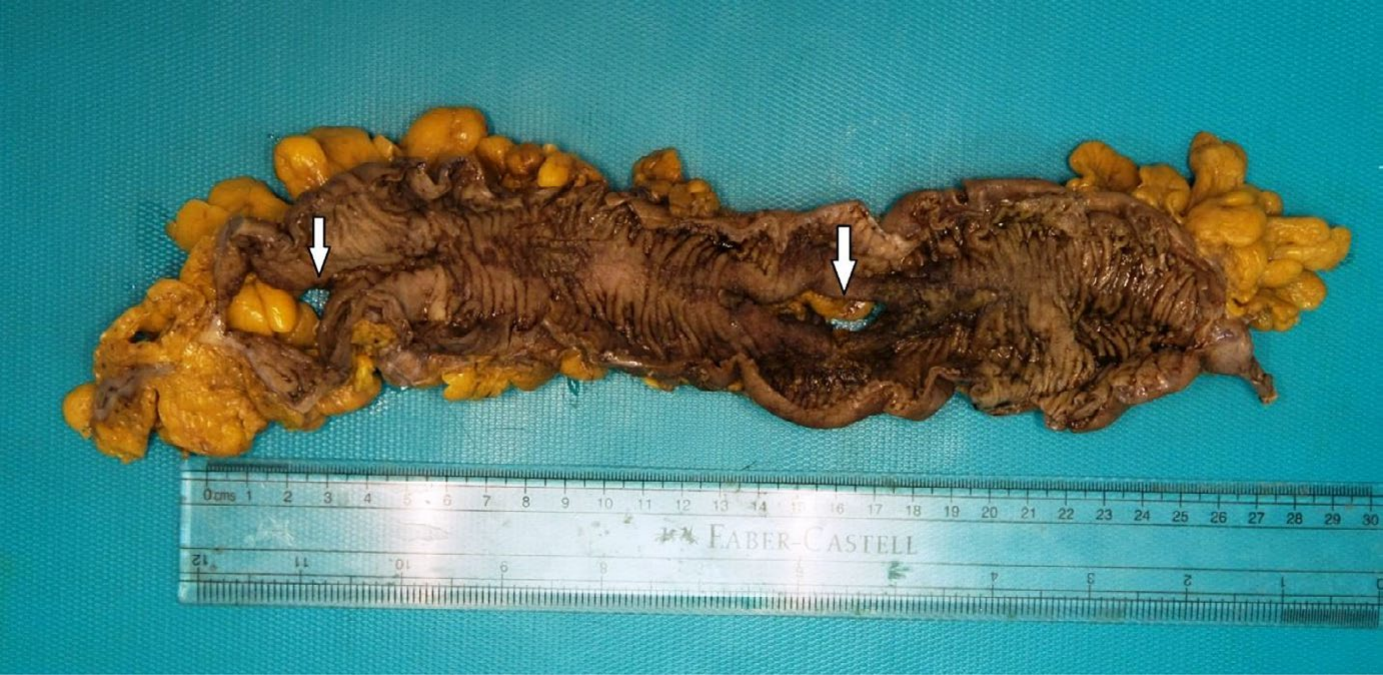
Gross photograph of the opened large bowel segment with two areas of full thickness perforation (arrows)

**Figure 2:**
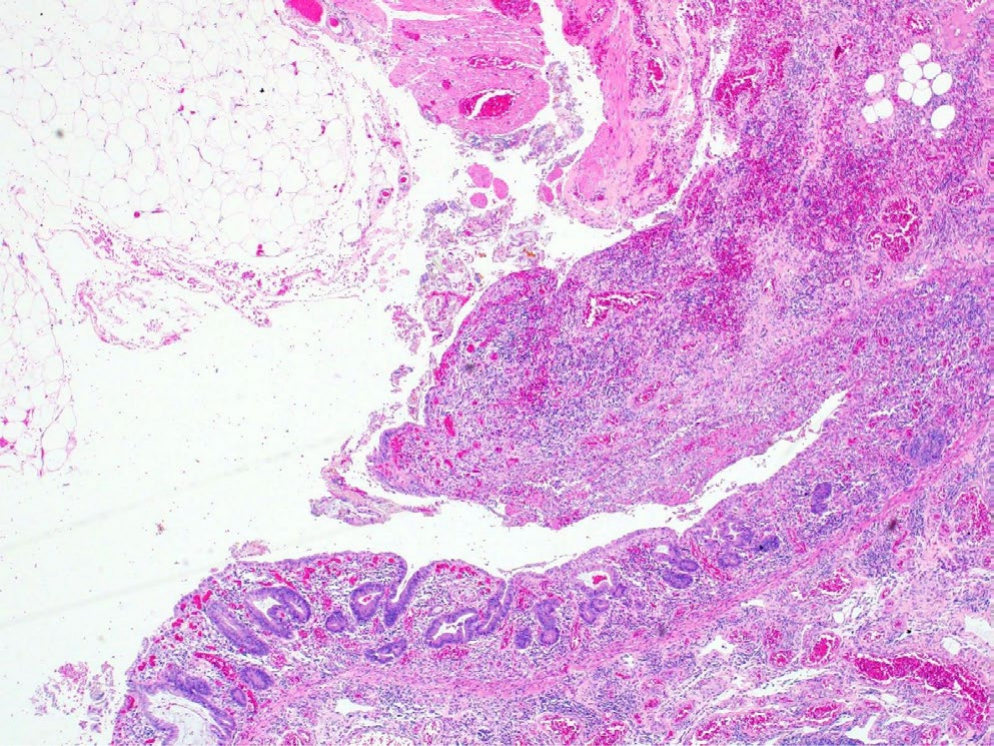
Low power magnification H&amp;E slide at the mucosal ulceration with mucosal sloughing and vascular congestion

**Figure 3:**
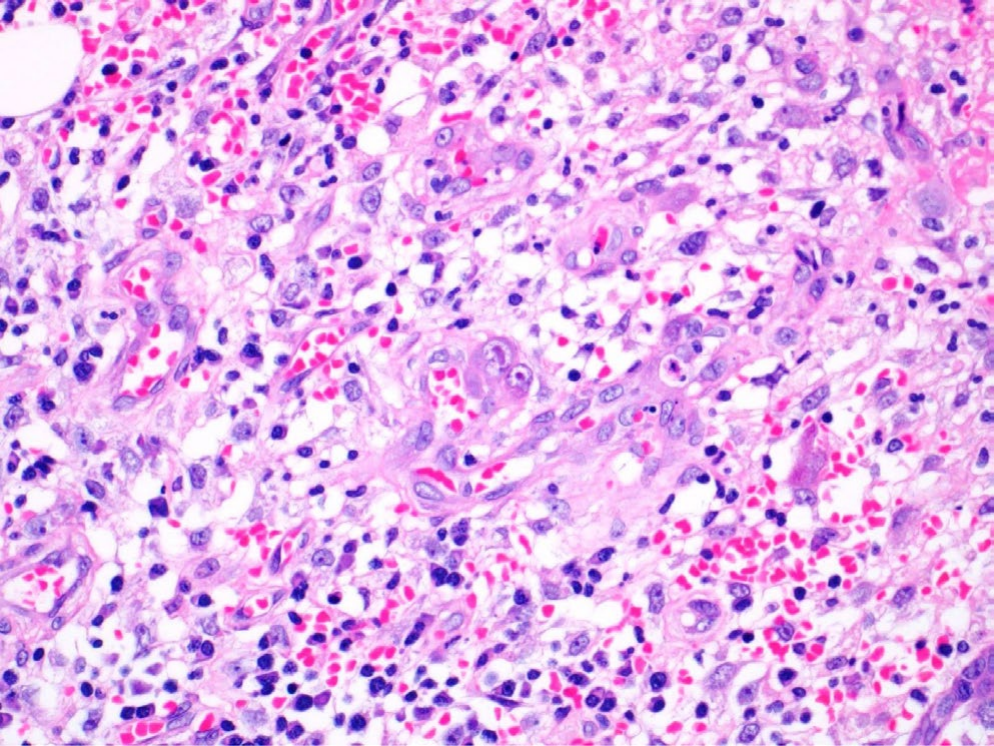
High power magnification photograph at the perforation site showing mixed acute and chronic inflammation in the lamina properia. In the middle of the photograph, there is enlarged cell with eosinophilic intranuclear and intracytoplasmic inclusions in the lamina properia in the ulcer bed, indicative of cytomegalovirus inclusions

**Figure 4:**
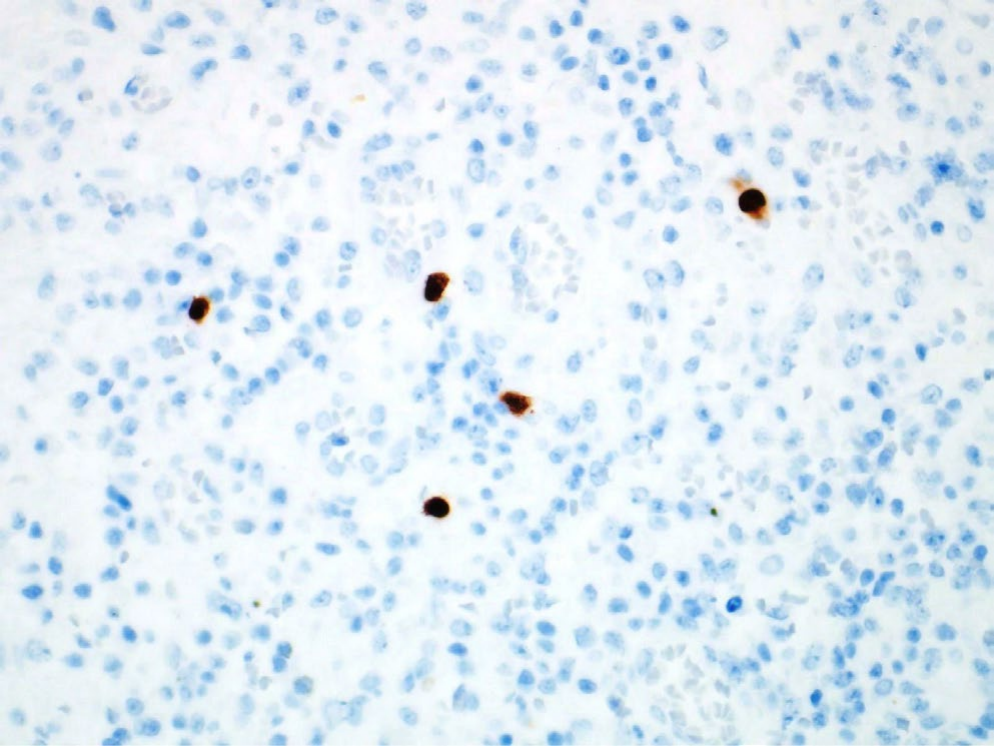
Cytomegalovirus immunohistochemical stain. The infected cells with CMV inclusions are highlighted in brown color

Cytomegalovirus (CMV) is a double‐stranded deoxyribonucleic acid virus belonging to the Herpesviridae family.^1^ It is more commonly seen in individuals with inflammatory bowel disease (IBD), hematological malignancies, organ transplantation, acquired immunodeficiency syndrome (AIDS), and patients on immunosuppressive therapy.^2^ The majority of primary CMV infections among immunocompetent people go not discovered.^3^ Nonspecific fever, occasionally accompanied by pancytopenia, characterizes uncomplicated CMV infection. Ulcerative changes can be seen when the colon becomes compromised by tissue‐invasive CMV. Watery diarrhea may begin to develop due to an inflammatory response, which might develop further into profuse bloody diarrhea. Subsequently, the colon may obstruct due to the development of inflammatory polyps. Severe inflammation and vasculitis may lead to perforation and peritonitis of the bowel due to ischemia and transmural necrosis.^4^ We describe a case of COVID‐19 pneumonia on immunosuppressive treatment presenting with life‐threatening lower gastrointestinal bleeding due to CMV colitis confirmed by serology and histopathology (Figures 1, 2, 3, 4).

## 2 CASE REPORT

**Figure 5:**
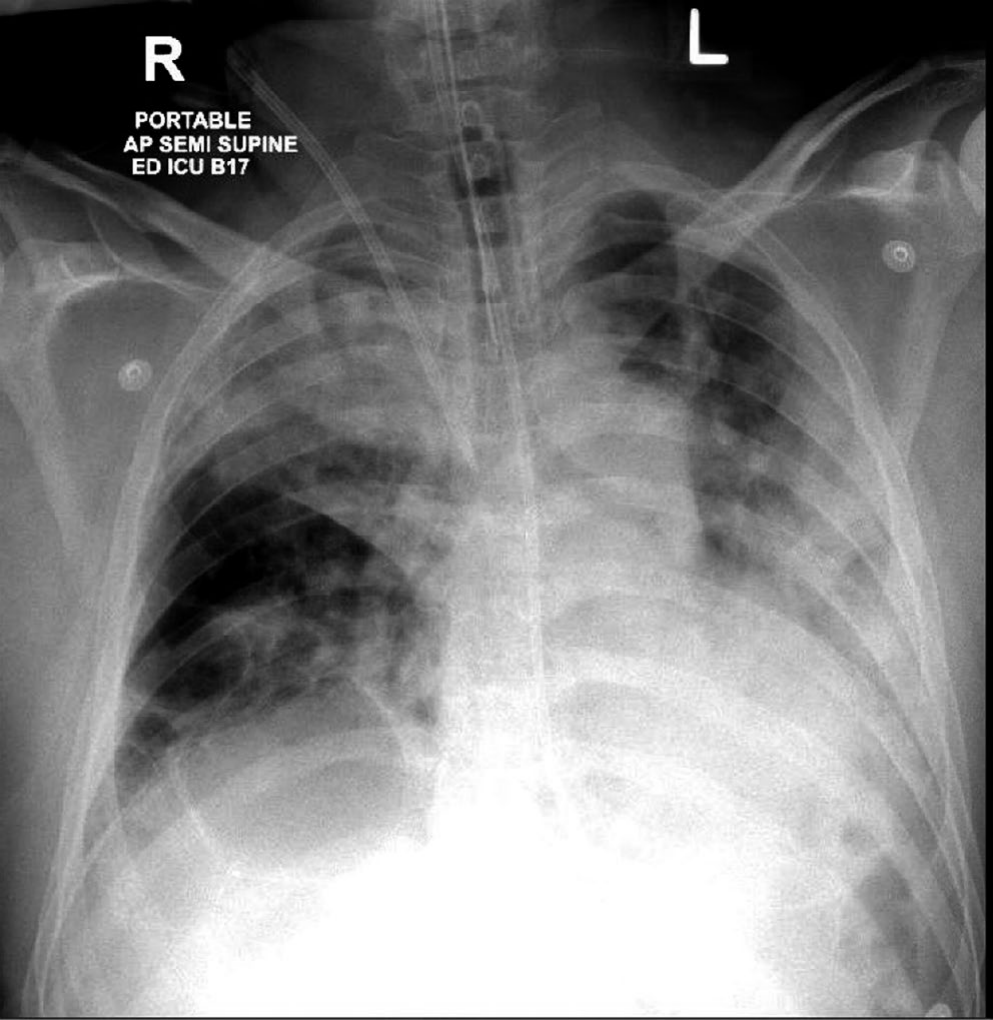
Shows bilateral consolidation of lung, endotracheal tube, nasogastric tube (NG Tube), right IJV (internal jugular vein), central venous catheter

A 42‐year‐old male patient with no past medical comorbidities was admitted to our Medical Intensive Care Unit (MICU) with a positive polymerase chain reaction (PCR) test for SARS‐CoV‐2 performed 7 days prior to admission. His presenting symptoms were fever, shortness of breath, cough, and generalized myalgia. For COVID‐19 pneumonia, he was initially supported with noninvasive ventilation (NIV) in a prone position. On day 2 of admission, there was a worsening of chest infiltrates with a spontaneous right pneumothorax for which a chest drain was placed. On the same day, he got intubated and proned for severe adult respiratory distress syndrome (ARDS) with a low Pao2/Fio2 ratio of 107 (Figure 5).

On day 3 of admission, he had a persistent unremitting fever, high ferritin levels (3584 ng/L), and high interleukin‐6 levels (176 pg/mL). There was strong suspicion for the presence of cytokine storm; hence, the patient was given an IL‐6 inhibitor, Tocilizumab (400mg).

On day 9, he had an upper gastrointestinal bleed (melena) with a drop in hemoglobin (Hb) from 10.5gm/dl to 9gm/dl. The gastroenterology team had managed him conservatively with proton pump inhibitors and by withholding low molecular weight heparin (LMWH). The melena subsided and Hb levels were stable. He received the second dose of tocilizumab 400 mg on the 16th day of ICU stay. He had another episode of melena with a significant drop in hemoglobin from 9 to 6 gm/dL on day 22 of admission. Esophagogastroduodenoscopy performed on the same day did not reveal any active bleeder or collection up to D3 of the duodenum. During this time, he was supported with multiple blood transfusions, and intravenous fluids, and the patient was stabilized hemodynamically.

He was tracheostomized due to prolonged intubation on day 28 of ICU stay. On the same day, he developed fresh bleeding per rectum with a drop in hemoglobin from 7.4 to 5.6 gm/dL. He was immediately taken for colonoscopy. The procedure was performed with great difficulty, and the scope could be advanced till the splenic flexure of the colon only. The colon was filled with blood clots, and fresh blood was noted circumferentially. The scope could not be passed further due to poor visibility owing to blood clots. Hemostasis could not be achieved, as no active bleeder was identified.

**Figure 6:**
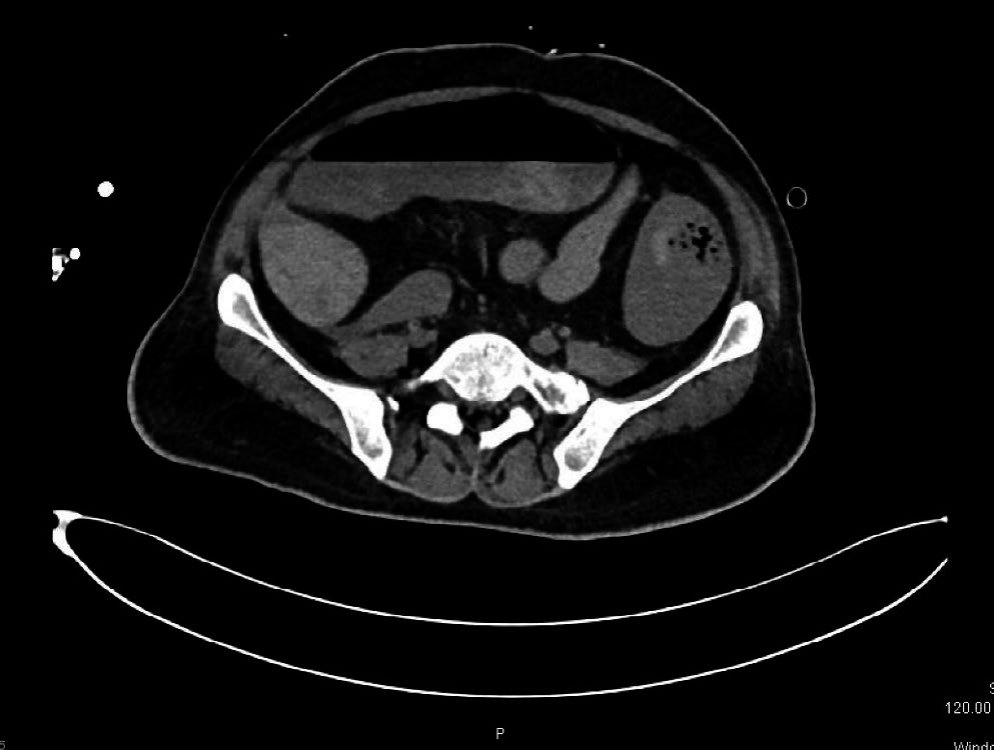
Mural thickening with intramural axial hemorrhage involving the splenic flexure of colon

A computed tomography (CT) angiogram was performed to identify a bleeder and for possible angioembolization. No bleeder could the, and the only positive finding was mural thickening with intramural axial hemorrhage involving the splenic flexure and proximal part of the descending colon (Figure 6). In view of hemodynamic instability and ongoing bleeding, a multidisciplinary team of surgeons decided to take him for life‐saving emergency laparotomy.

On the table, colonoscopy was performed through the enterostomy site and passed the scope up to three meters of the small bowel, no bleeding site was identified, and enterostomy was closed. Left hemicolectomy was done in view of the suspicious site of bleeding at the splenic flexure, and double‐barrel stoma was created. The histopathology of the resected colon was reported as CMV colitis with colonic perforation and serology confirmed the CMV infection. He was then started on Ganciclovir therapy 220 mg once daily for 4 days, followed by 440mg every 12 hours for 3 weeks. Timely diagnosis and performance of life‐saving surgery helped our patient's survival. During the MICU stay, he received hydroxychloroquine, antibiotics, antifungals, and methylprednisolone for COVID‐19 ARDS. The patient was successfully discharged to the inpatient ward with a functional colostomy on day 50. On day 61, SARS‐CoV‐2 PCR was negative, and the patient was discharged to a rehabilitation center.

## 3 DISCUSSION

CMV infections are becoming more prevalent in immunocompetent patients.^5^ Recently, with the outbreak of COVID 19 disease, many studies have suggested that COVID‐19 may activate dysregulated host immune responses, wherein interleukin‐6 (IL‐6) levels are elevated in cases of severe COVID‐19. The role of antihuman interleukin‐6 receptor (IL‐6R) monoclonal antibody such as tocilizumab may be beneficial.^6^ However, the available evidence is still inconclusive of its routine use against COVID 19.

Al‐Omari et al noted that the risk factors such as immunocompromised state, mechanical ventilation, and sepsis were found to be strongly associated with CMV. In contrast, the use of corticosteroids, blood transfusion, and stress was weakly associated. They found no association with risk factors such as age, gender, disease severity scores, and active malignancy.^7^

CMV demonstrates an organ‐specific tropism within the body, affecting mainly hemopoietic stem cells and parenchymal connective tissue. As per the systemic review done by Rafailidis et al, in immunosuppressed patients with tissue‐invasive (TI) CMV, the gastrointestinal tract is the most commonly affected and comprises almost 30% of the disease.^5^ CMV lesions may be present throughout the gastrointestinal tract, from the oral cavity to the rectum. However, colon involvement is the most common, comprising up to 94% of cases.^8^, ^9^, ^10^ For viral reactivation in the immunocompetent individuals, critical illness is a major predisposing factor for developing TI‐CMV disease as in our case.^11^

IL‐6 has proinflammatory and antiviral properties. With IL‐6 antagonist therapy reactivation of CMV, EBV is a potential concern. Other opportunistic infections like candida, Pneumocystis jirovecii pneumonia, Herpes zoster, EBV hepatitis, Tuberculosis, and asymptomatic Mycobacterium avium‐intracellulare have been reported in clinical trials.^12^

Van duin et al reported a case of CMV reactivation with pneumonitis while being treated with tocilizumab for rheumatoid arthritis.^12^ In a patient being treated with tocilizumab for rheumatoid arthritis, acute hepatitis and gastric erosions induced by CMV reactivation were reported by Kamura et al.^13^

The presence of CMV infection in our patient was diagnosed based on the presence of inclusion bodies on histopathology and subsequently confirmed by viral serology. Biopsy with immunohistochemistry using monoclonal antibodies against CMV antigens is now considered the gold standard.^14^ The characteristic Owl's eye inclusion bodies are highly specific for CMV.^15^ The human blood‐derived products (convalescent plasma, immunoglobulin) and immune‐modulatory therapies, like tocilizumab and corticosteroids, studied extensively for the treatment of severe SARS‐CoV‐2 infection. In the RECOVERY trial,^16^ dexamethasone therapy has shown a mortality benefit among COVID 19 patients who were requiring supplemental oxygen. Although the risk associated with steroid therapy is weak, it cannot be completely ruled out in the occurrence of CMV infection.

We suggest high‐risk patients receiving tocilizumab should be screened for viral reactivation and other opportunistic infections.

## 4 CONCLUSION

This case highlights the importance of keeping a high suspicion and diagnosing the pathology at the earliest to treat and control the disease in these critically ill patients. Though they may be immunocompetent with no risk factors prior to admission to the ICU, they become vulnerable, and the risk of developing primary/latent CMV infection is high. A timely diagnosis can prevent prolonged ICU and hospital stay. It also warrants judicious use of corticosteroids and other immunomodulatory drugs like tocilizumab in critically ill patients. In the present scenario, while treating patients with COVID‐19, the use of immunosuppressive therapy should be justified while being alert for signs and symptoms of CMV reactivation and other opportunistic infections. Early recognition of CMV infection and initiation of early treatment will prevent life‐threatening bleeding and mortality.

## CONFLICT OF INTEREST

None declared.

## AUTHOR CONTRIBUTIONS

MYK, KSS, AAA, MAA, AJN, ASM: involved in data collection, literature search, and manuscript preparation. MZS and BKA: involved in histopathology slides and manuscript preparation. All authors read and approved the final manuscript.

## ETHICS APPROVAL AND CONSENT TO PARTICIPATE

The article describes a case report. Therefore, no additional permission from our Ethics Committee was required.

## CONSENT FOR PUBLICATION

The consent for publication was obtained.

## Data Availability

All data generated or analyzed during this study are included in this published article.
